# Reaction Mechanism for CO Reduction by Mo-Nitrogenase Studied by QM/MM

**DOI:** 10.1021/acs.inorgchem.4c02323

**Published:** 2024-08-14

**Authors:** Hao Jiang, Ulf Ryde

**Affiliations:** Department of Computational Chemistry, Lund University, Chemical Centre, P.O. Box 124, SE-221 00 Lund, Sweden

## Abstract

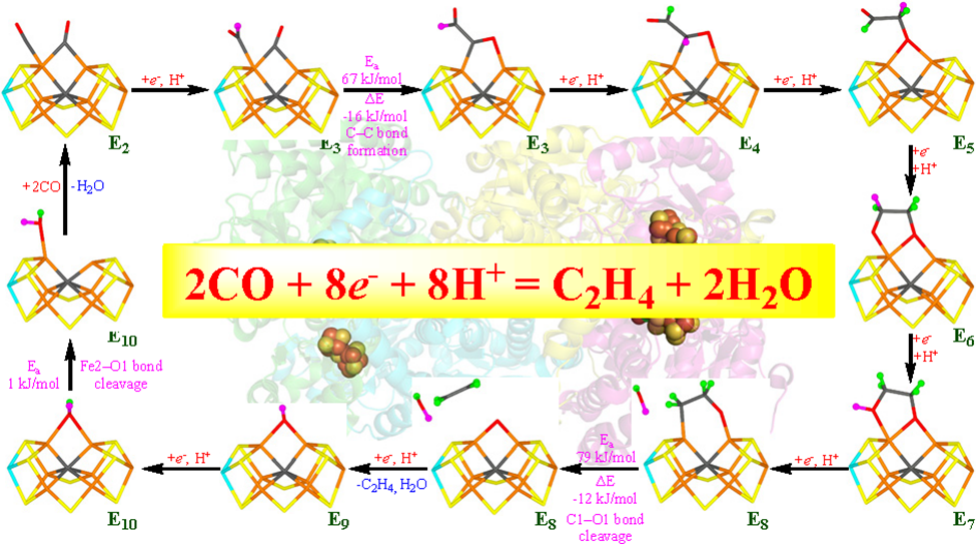

We have studied the conversion of two molecules of carbon monoxide to ethylene catalyzed by nitrogenase. We start from a recent crystal structure showing the binding of two carbon monoxide molecules to nitrogenase and employ the combined quantum mechanics and molecular mechanics approach. Our results indicate that the reaction is possible only if S2B dissociates as H_2_S (i.e., the charge of the FeMo cluster remains the same as in the E_0_ state, indicating that the Fe ions are formally reduced two steps when CO binds). Eight electrons and protons are needed for the reaction, and our mechanism suggests that the first four bind alternatively to the two carbon atoms. The C–C bond formation takes place already after the first protonation (in the E_3_ state). The next two protons bind to the same O atom, which then dissociates as water. In the same state (E_8_), the second C–O bond is cleaved, forming the ethylene product. The last two electrons and protons are used to form a water molecule that can be exchanged by S2B or by two CO molecules to start a new reaction cycle.

## Abstract 2

Nitrogenase can convert carbon monoxide into ethylene. Based on recent crystal structures and QM/MM calculations, we studied this reaction involving eight electrons and protons. The results indicate that the C−C bond forms in the E_3_ state and ethylene forms in the E_8_ state.

## Introduction

Nitrogenase (EC 1.18/19.6.1) is the only enzyme that can break the N–N bond in N_2_ and convert it into ammonia.^1^ It is found in a few groups of bacteria and functions at ambient temperature and pressure. Crystallographic studies have revealed that the most active type of nitrogenase consists of two proteins. The MoFe protein contains the catalytic MoFe_7_S_9_C(homocitrate)CysHis cluster (FeMo cluster) and the electron-transfer Fe_8_S_7_Cys_6_ cluster (P cluster), whereas the Fe protein contains a Fe_4_S_4_ cluster. There are also alternative nitrogenases that have V or Fe in place of Mo ion, but they exhibit lower activity toward N_2_.^1−3^

Carbon monoxide (CO) can both inhibit or act as a substrate for nitrogenase. CO is a colorless and toxic gas that is isoelectronic with N_2_. Nitrogenase has been found to reduce CO to hydrocarbons, a process that is an important alternative to the industrial Fischer–Tropsch process.^4−6^ Several groups have studied the binding of CO to nitrogenase. The presence of carbon monoxide produces two distinctive characteristic *S* = 1/2 EPR signals, called loCO and hiCO, which arise under low and high CO pressure, respectively.^7,8^ One or two CO molecules have been confirmed to be bound to the FeMo cluster at low and high CO pressure, respectively, through Fe electron nuclear double resonance (ENDOR) measurements.^9,10^ The symmetry of the hyperfine coupling tensor extracted from ^13^C ENDOR spectra suggests that the loCO species contains a single bridging μ-CO ligand, while the hiCO species contains two terminal CO ligands.^11^ Another species of MoFe protein possessing two CO molecules was observed through EPR spectroscopy under high electron flux (with an *S* value of 3/2) and given the name hi(5)-CO.^12^

**Figure 1:**
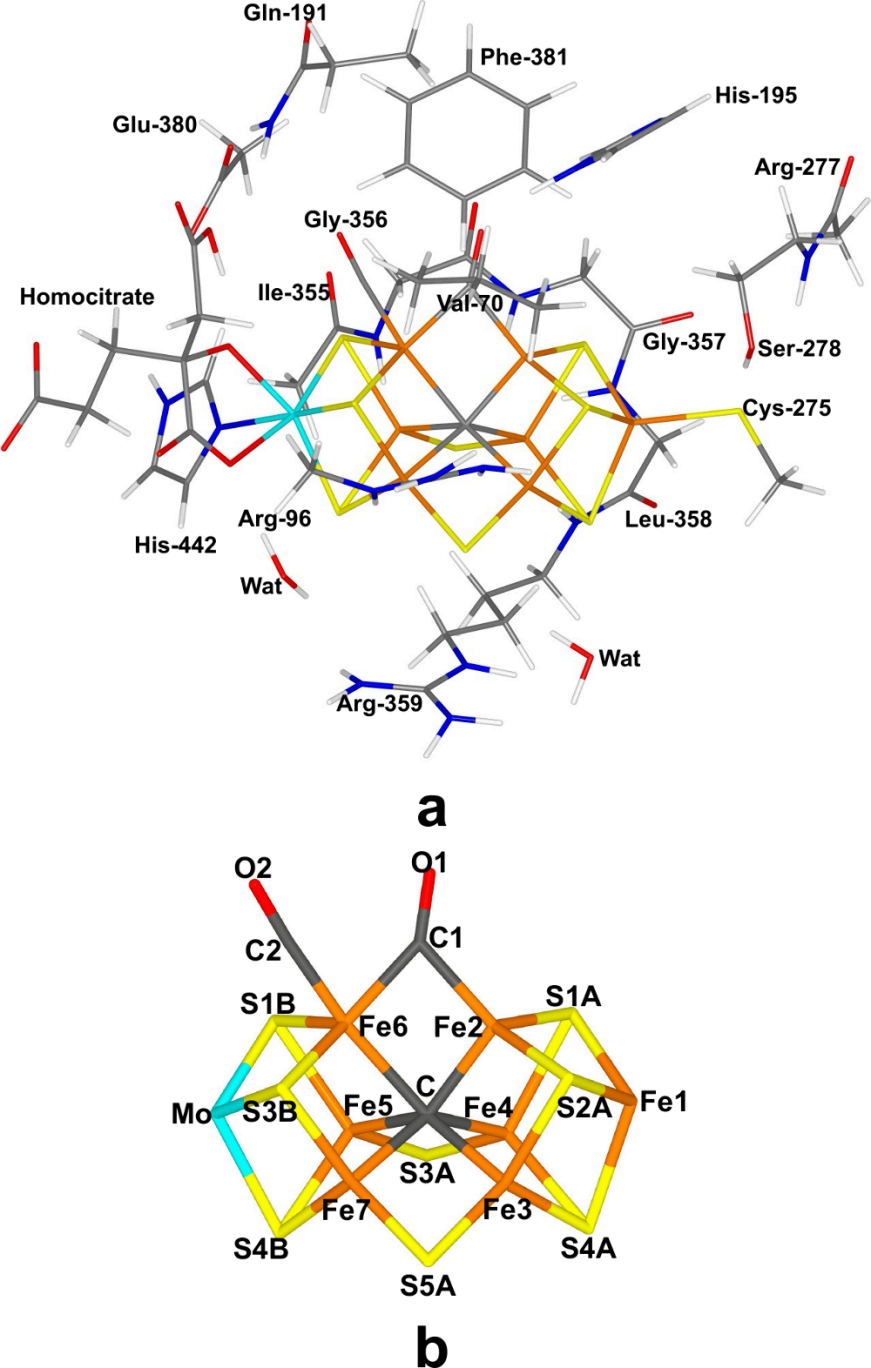
(a) Structure of the FeMo cluster also illustrating the QM system used in all calculations, as well as the names of the nearby residues. (b) The lower figure shows only the FeMo cluster with atom names indicated. Both figures show the E_2_ state with two bound CO molecules.

A crystal structure reported by Spatzal et al. (corresponding to loCO) provides insight into the binding mode of CO.^13^ They found that the FeMo cluster had a bridging CO ligand that replaced the S2B belt sulfide ion between the Fe2 and Fe6 atoms. Recently, Spatzal et al. reported a crystal structure that shows two CO ligands coordinated to the FeMo-cofactor of Mo nitrogenase at a resolution of 1.33 Å.^12^ One CO molecule bridges Fe2 and Fe6 as in the other crystal structure, whereas the second CO ligand binds terminally to Fe6 (Figure 1). Einsle et al. also reported two CO-binding sites in V nitrogenase at the same time, with binding modes that are strikingly similar to those of Mo-nitrogenase.^14^ They suggested a reaction mechanism for CO reduction to ethylene, involving the repeated insertion of hydride ions, terminally bound to Fe6, into the substrate bridging Fe2 and Fe6.^15^ Likewise, a second CO molecule terminally bound to Fe6 could react with a methyl radical, forming the C–C bond. Thus, the mechanism involves intermediates at the formyl, methanol, methyl, acetyl, ethanol, and ethyl levels. Finally, ethylene is formed by β-elimination of a hydride ion.

After revisiting the activity of the Mo nitrogenase toward CO reduction, Ribbe and co-workers found that it can reduce and couple CO to C2–C4 hydrocarbons, including C_2_H_4_, C_2_H_6_, C_3_H_6_, and C_3_H_8_, whereas CH_4_ was not detected.^16^ Ethylene is the simplest alkene and an important raw material for chemical industry.^17^ It is also an important natural plant hormone.^18^ In the context of combating global warming and promoting low-carbon lifestyles, the conversion of CO to C_2_H_4_ holds significant potential for reducing greenhouse gas emissions.

This reaction catalyzed by nitrogenase has not been much investigated with computational methods. The lack of crystal structures of the carbon monoxide binding to the nitrogenase active site has been a limiting factor for early studies of this reaction. For example, Dance studied V-nitrogenase,^19^ which also reduces CO to hydrocarbons, but the calculations involved a model with a central nitrogen atom (instead of carbon) and no carbonate ligand.

Here, we use the crystal structure with two molecules of carbon monoxide to study the formation of ethylene by Mo nitrogenase, using the combined quantum mechanical and molecular mechanics (QM/MM) approach. Given the rapid nature of biological enzyme reactions, it is often challenging to capture reaction intermediates using existing experimental techniques, making computational simulation methods a powerful tool for investigating enzyme reaction mechanisms. Our work provides new insights into the mechanism of carbon monoxide reduction by nitrogenase.

## Methods

### Protein

The calculations were based on the 1.0 Å crystal structure of the Mo nitrogenase from *Azotobacter vinelandii* (PDB code 3U7Q).^20^ The setup of the protein was identical to that of our previous studies.^21,22^ The entire heterotetramer was considered in the calculations because the various subunits are intertwined and cannot be easily separated. The quantum mechanical (QM) calculations focused on the FeMo clusters in the C subunit because there is a solvent-derived imidazole molecule close to the active site (∼11 Å) in the A subunit. The two P-clusters and the FeMo cluster in subunit A were modeled using molecular mechanics (MM) in the fully reduced and resting states, respectively, using a QM charge model.^21^

The protonation states of all residues were the same as before:^21^ All Arg, Lys, Asp, and Glu residues were assumed to be charged, except Glu-153, 440, and 231D (a letter “D” after the residue number indicates that it belongs to that subunit; if no letter is given, it belongs to subunit C; subunits A and B are identical to the C and D residues). Cys residues coordinating to Fe ions were assumed to be deprotonated. His-274, 451, 297D, 359D, and 519D were assumed to be protonated on the ND1 atom, His-31, 196, 285, 383, 90D, 185D, 363D, and 457D were presumed to be protonated on both the ND1 and NE2 atoms (and therefore positively charged), whereas the remaining 14 His residues were modeled with a proton on the NE2 atom. The homocitrate ligand was modeled in the singly protonated state with a proton shared between the hydroxy group (which coordinates to Mo) and the O1 carboxylate atom. This protonation state was found to be the most stable one in an extensive QM/MM, molecular dynamics, and quantum-refinement study and was is also supported by another QM/MM study.^23^

The protein was solvated in a sphere with a radius of 65 Å around the geometrical center of the protein. 160 Cl^–^ and 182 Na^+^ ions were added at random positions (but not inside the protein) to neutralize the protein and give an ionic strength of 0.2 M.^24^ The final system contained 133 915 atoms. The added protons, counterions and water molecules were optimized by a simulated annealing calculation (up to 370 K), followed by a minimization, while holding the other atoms fixed at the positions determined by the crystal structure.^21^

All MM calculations were performed with the Amber software.^25^ The Amber ff14SB force field^26^ was used for the protein, and water molecules were described using the TIP3P model.^27^ The metal sites were treated by a nonbonded model^28^ and charges were obtained with the restrained electrostatic potential method at the TPSS/def2-SV(P) level of theory^29,30^ and sampled with the Merz–Kollman scheme.^31^ The same MM parameters were used for the metal sites as in previous investigations.^21^

The FeMo cluster was modeled by MoFe_7_S_8_C(homocitrate)(CH_3_S)(imidazole), where the two last groups are models of Cys-275 and His-442. In addition, all groups that form hydrogen bonds to the FeMo cluster were also included in the QM model (viz., Arg-96, Gln-191 and His-195 (side chains); Ser-278 and Arg-359 (both backbone and side chain, including the CA and C and O atoms from Arg-277); Gly-356, Gly-357, and Leu-358 (backbone, including the CA, C and O atoms from Ile-355), as well as two water molecules). The side chain of Glu-380 was also included because it forms hydrogen bonds with Gln-191 and His-442, as well as the side chains of Val-70 and Phe-381 because they are close to Fe2 and Fe6, the reactive Fe ions. Following the recent crystal structure of Mo-nitrogenase and V-nitrogenase with two CO bound,^12^ S2B was removed from the FeMo cluster. The QM system involved 191–199 atoms in total (depending on the number of added protons) and it is shown in Figure 1.

### QM Calculations

All QM calculations were performed with the Turbomole software (version 7.5).^32^ All structures were studied with the r^2^SCAN^33^ functional and the def2-SV(P)^30^ basis set. The calculations were sped up by expanding the Coulomb interactions in an auxiliary basis set, the resolution-of-identity (RI) approximation.^34,35^ Empirical dispersion corrections were included with the DFT-D4 approach,^36^ as implemented in Turbomole. All minima were fully optimized without any restraints. Approximate transition states were obtained as the highest point on the relaxed potential energy surface along a reaction coordinate (e.g., C–C, C–H, or O–H distances), which were scanned with a step of 0.1 Å near the transition states.

Based on the EPR experiments, discussed in the Introduction, we used the doublet spin state for the starting structure of nitrogenase with two CO molecules bound. For the other *E*_*n*_ states, we studied which of the two or three lowest spin states had the most favorable energy at the r^2^SCAN/def2-SV(P) level of theory.

The electronic structure of all QM calculations was obtained with the broken-symmetry (BS) approach:^37^ Each of the seven Fe ions was modeled in the high-spin state, with either a surplus of α (four Fe ions) or β (three Fe ions) spin. Such a state can be selected in 35 different ways .^38^ The various BS states were obtained either by swapping the coordinates of the Fe ions^39^ or with the fragment approach by Szilagyi and Winslow.^40^ The various BS states are named by listing the number in the Noodleman nomenclature (BS1–10),^37^ followed by the numbers of the three Fe ions with minority spin (in the tables, only the latter three numbers are given). All structures were first studied in BS10–147 state (i.e., with minority spin on Fe1, Fe4, and Fe7). Then, we did a comprehensive study of all BS states for the lowest structure and used the best BS state found for all structures.

### QM/MM Calculations

QM/MM calculations were performed with the ComQum software.^41,42^ In this approach, the protein and solvent are split into two subsystems: System 1 (the QM region) was relaxed by QM methods. System 2 contained the remaining part of the protein and the solvent, and it was kept fixed at the original coordinates (equilibrated crystal structure) to avoid the risk that different calculations end up in different local minima. The total system was spherical and nonperiodic with 133 915 atoms.

In the QM calculations, system 1 was represented by a wave function, whereas all the other atoms were represented by an array of partial point charges, one for each atom, taken from the MM setup. Thereby, the polarization of the QM system by the surroundings is included in a self-consistent manner (electrostatic embedding). When there is a bond between systems 1 and 2 (a junction), the hydrogen link-atom approach was employed: The QM system was capped with hydrogen atoms (hydrogen link atoms, HL), the positions of which are linearly related to the corresponding carbon atoms (carbon link atoms, CL) in the full system.^41,43^ All atoms were included in the point-charge model, except the CL atoms.^44^

The total QM/MM energy in ComQum is calculated as^41,42^
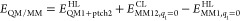
1where *E*_QM1+ptch2_^HL^ is the QM energy of the QM system truncated by HL atoms and embedded in the set of point charges modeling system 2 (but excluding the self-energy of the point charges).  is the MM energy of the QM system, still truncated by HL atoms, but without any electrostatic interactions. Finally,  is the classical energy of all atoms in the system with CL atoms and with the charges of the QM region set to zero (to avoid double-counting of the electrostatic interactions). Thus, ComQum employs a subtractive scheme with van der Waals link-atom corrections.^45^ No cutoff is used for any of the interactions in the three energy terms in eq 1.

The geometry optimizations were continued until the energy change between the two iterations was less than 2.6 J/mol (10^–6^ a.u.) and the maximum norm of the Cartesian gradients was below 10^–3^ a.u.

QM/MM calculations give comparable energies only if they contain the same number of electrons and atoms of each element in both the QM and MM systems. Therefore, we compare only structures within the same E_*n*_ level. For each transition from E_*n*_ to E_*n*+1_, an electron and a proton are added to the QM system, and we compare energies of structures with this proton in different positions and with different intermediates formed from the two CO molecules.

## Result and Discussion

In this study, we investigate the CO-reduction reaction mechanism of nitrogenase, starting from a crystal structure with two CO molecules bound to the FeMo cluster.^12^ CO binds only to reduced states of nitrogenase and at high CO pressure, it is normally assumed that it binds to the doubly reduced E_2_ state.^14,15,46,47^ It is widely believed that for each electron taken up by the FeMo cluster, also a proton is taken up. Therefore, the net charge of the FeMo cluster remains the same for all E_*n*_ states. Thus, the E_2_ state (i.e., with two electrons and protons more than E_0_) has the same charge as the E_0_ state (−4 for our QM system). If we use MFC to denote the MoFe cluster without S2B and in the standard Fe_3_^II^Fe_4_^III^ oxidation state of E_0_ and use the net charge of our QM system, then we can write the reaction from E_0_ to E_2_ as [MFC(S2B^2–^)]^4–^ + 2*e*^–^ + 2H^+^ → [MFC(S2B^2–^, 2*e*^–^, 2H^+^)]^4–^). Upon CO binding, the S2B sulfide ligand is displaced. Quantum refinement of a CO-bound crystal structure indicated that no protons remain in the cluster.^48^ This gives two possible charge states of the (CO)_2_-bound E_2_ state, either −2 (if the two protons have dissociated as H_2_ (i.e., the reaction [MFC(S2B^2–^, 2*e*^–^, 2H^+^)]^4–^ + 2CO → [MFC(CO)]^2–^ + H_2_ + S2B^2–^; in this case the oxidation state of the Fe ions remains the same as in the E_0_ state, Fe_3_^II^Fe_4_^III^) or −4 if they instead dissociate together with S2B as SH_2_, (i.e., the reaction [MFC(S2B^2–^, 2*e*^–^, 2H^+^)]^4–^ + 2CO → [MFC(CO), 2*e*^–^]^2–^ + H_2_S2B) leading to the same net charge of the QM system as in the E_0_ state but with a formal double reduction of the FeMo cluster (to Fe_5_^II^Fe_2_^III^). In this investigation, we have tested both these charge states.

The main product of the CO reaction is C_2_H_4_.^16^ Therefore, we study the reaction:
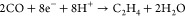
2

In analogy with the normal reaction on nitrogenase, we call the various intermediates in the reaction mechanism E_*n*_, where *n* indicates the number of added electrons and protons compared to the resting E_0_ state. We discuss states at different oxidation levels in separate sections, from E_2_ to E_10_.

### E_2_ Structures with Two CO Molecules Bound

We start with the E_2_ state with two bound CO molecules. This state is known from a crystal structure, showing that one CO molecule replaces the S2B ligand (bridging Fe2 and Fe6), whereas the second CO molecule binds end-on to Fe6.^12^ We denote the atoms of these two CO molecules C1–O1 and C2–O2 as shown in Figure 1b.

In the optimized structure of the −4 charge state, the Fe2–C1 and Fe6–C1 distances are 1.86 and 1.89 Å, respectively, which are in reasonable agreement with those found in the crystal structure (1.93 and 1.92 Å).^12^ The same applies to the C2–O2 bond length, 1.16 and 1.12 Å, respectively. However, the optimized Fe6–C2 distance, 1.74 Å, is much shorter than the corresponding distance in the crystal structure (2.03 Å). Likewise, the C1–O1 bond length, 1.21 Å, is much longer than in the crystal structure 0.95 Å (but similar to the C2–O2 bond length). The other charge state (−2) gives nearly identical bond lengths, within 0.04 Å for the reported bonds. To further check the reliability of the r^2^SCAN structures, we optimized the same structure also with three other functionals, TPSS, TPSSh, and B3LYP. The results are listed in Table S1 and are shown Figure S1. They all support the r^2^SCAN results, giving C1–O1 bond lengths of 1.18–1.19 Å and Fe6–C2 distances of 1.77–1.80 Å. For the 11 distances in Table S1, all four DFT functionals give a mean absolute deviation to the crystal structure of 0.11–0.14 Å, but only 0.03–0.06 Å to the r^2^SCAN structure. Likewise, an r^2^SCAN structure using another spin (quartet) and BS state (BS10–135) gives distances that agree within 0.02 Å on average. Consequently, we believe that the details of the QM/MM structures are more accurate than the crystal structure.

The bridging CO group forms a hydrogen bond to His-195 with a O1–H distance of 1.62 Å (−4 charge state; cf. Figure S1b). One of the HE2 protons of Gln-191 point toward O2 of the terminal CO group, but the O2–HE2 distance is rather large, 2.35 Å. In fact, the same proton is closer to the O1 atom of homocitrate, 2.30 Å. While it has been documented that Gln-191 sometimes flips to another conformation in some crystal structures in which S2B dissociates,^49^ no such behavior was observed in the presence of two carbon monoxide ligands. We also tested a flipped conformation of Gln-191, but such a structure was 106 kJ/mol less stable than the nonflipped state (shown in Figure S1d).

We studied E_2_ in the doublet state and did a full BS states investigation which indicated that BS10–147 is the best state. Therefore, we used this BS also for other structures. However, the quartet state is close in energy (only 1 kJ/mol less stable in the BS10–147 state). We also tested another protonation state for His-195 (protonated on ND1, instead of NE2), but this structure was 68 kJ/mol higher in energy. Moreover, we tried to form a C–C bond between the two CO molecules, and we can get such a structure (i.e., with acetylendiolate), but it is 91–98 kJ/mol less stable than the structure without the C–C bond for the two charge states. Finally, we attempted to transfer a proton from homocitrate to C2 and O2, but such transfers were strongly unfavorable.

### E_3_ Structures

**Figure 2:**
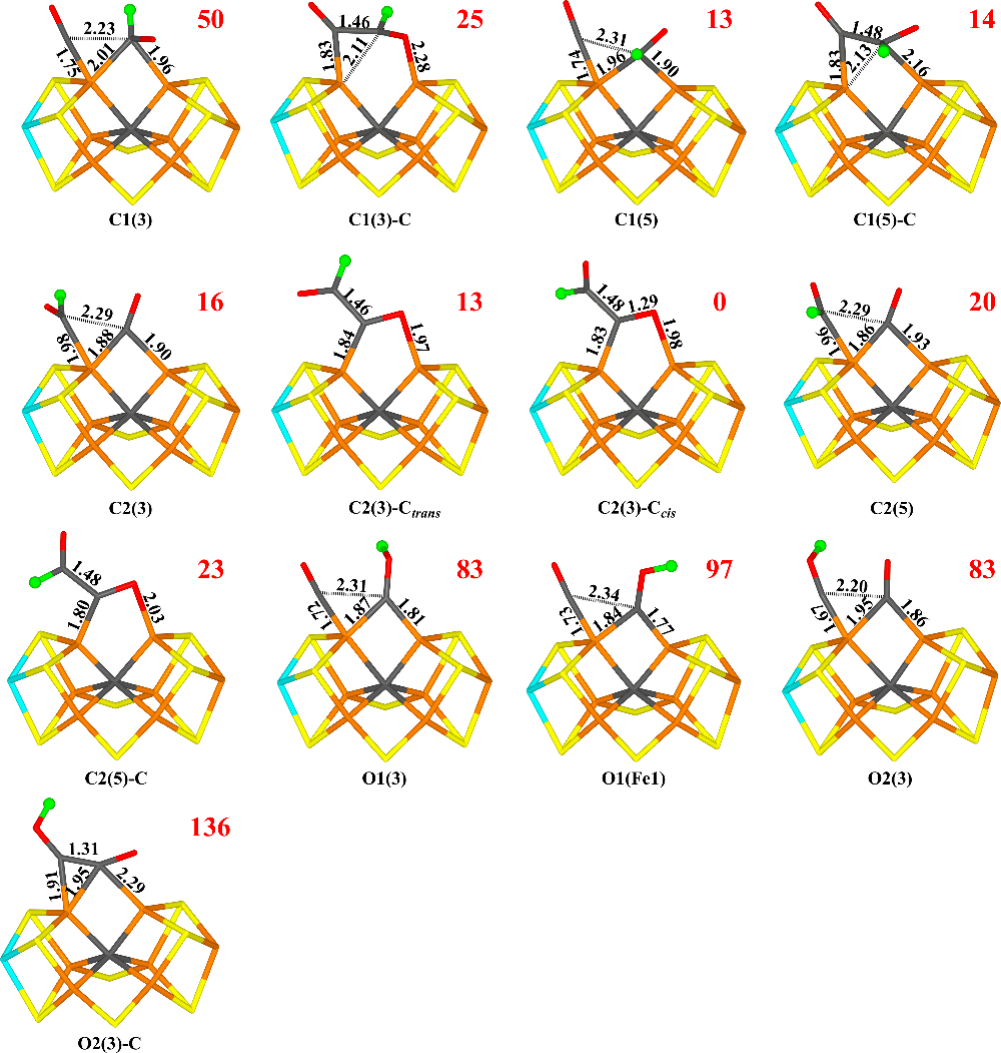
Structures considered for the E_3_ state, showing those obtained for the −4 charge state. The added proton is shown as a green ball. Relative energies (kJ/mol) for the −4 charge states are indicated for each structure.

Next, we added an electron and a proton to the FeMo cluster to obtain structures at the E_3_ level. We tried to add the proton to all four substrate atoms, C1, C2, O1 and O2, and for most of them, we tested two different directions of the proton, viz. pointing either toward S3A or S5A. The structures are named after the protonated atom with the direction indicated with the atom number (S**3**A or S**5**A) in brackets (e.g., C1(3) or C1(5)). We also tried to form a C1–C2 bond for most structures. For such structures, “-C” is appended (e.g., C2(3)-C). The tested structures are shown in Figure 2. We first optimized all structures in the BS10–147 state. For the structure with the lowest energy, we studied all 35 BS states. BS10–125 was found to be lowest in energy. Therefore, all structures were reoptimized for the BS10–125 state, but they often changed to the BS8–347 state, because the spin population on Fe6 is low and often changes sign during the geometry optimization. We studied structures both in the −2 and −4 charge states. We tested three spin states, *S* = 0, 1, or 2, and found that the singlet state is most stable. The obtained structures are listed in Table 1 and are shown in Figure 2.

**Table 1:** Relative Energies (kJ/mol) of the Various Structures Studied for the E_3_ State, Using Two Different Total Charges of the QM Region, – 4 or −2^a^

Structure	–4	–2
C1(3)	50^b^	16^c^
C1(3)-C	25	44^c^
C1(5)	13	20^c^
C1(5)-C	14	29^c^
C2(3)	16	31^c^
C2(3)-C_*trans*_	13	2
C2(3)-C_*cis*_	0	0
C2(5)	20	42^c^
C2(5)-C	23	23^c^
O1(3)	83	40^c^
O1(Fe1)	97	54^c^
O2(3)	83	136^c^
O2(3)-C	136	91^c^

a All structures were studied in the singlet BS8-347 state, if not otherwise stated.

b BS10–125.

c BS10–147.

The most favorable structure for both charge states is C2(3)-C with a C–C bond formed (i.e., deprotonated glyoxal, OCHCO^–^). The C–C bond length is 1.48 and 1.50 Å in the −4 and −2 charge states, respectively. C1 coordinates to the FeMo cluster in an asymmetric bridging mode with C1–Fe6 and C1–Fe2 distances of 1.83 and 2.38 Å (1.86 and 2.37 Å). This brings the O1 atom close to Fe2 with a O1–Fe2 distance of 1.98 (1.97) Å. There is a significant spin population on C1 (0.25–0.31 *e*), indicating some radical character. O1 receives a hydrogen bond from HE2 of His-195 (1.81 or 1.87 Å).

In fact, when a C–C bond is formed, the CHO group aligns with the other CO group and the difference between the two directions of the added proton becomes less pronounced. Instead, the structures differ more in whether the two O atoms are in *cis* or *trans* positions to each other. The most favorable structure (C2(3)-C_*cis*_) has them in a somewhat twisted *cis* position (the O1–C1–C2–O2 dihedral angle is −30°). Another structure, which was also obtained from C2(3) (called C2(3)-C_*trans*_ in Table 1 and Figure 2), instead has the two O atoms in *trans* positions (the O1–C1–C2–O2 dihedral angle is −174°) is 13 kJ/mol less stable (2 kJ/mol for the −2 charge).

In the −4 charge state, six additional structures have a similar stability as C2(3)-C_*trans*_ (within 13 kJ/mol). These include C1(5), C1(5)-C, C2(3), C2(5), C2(5)-C, and C1(3)-C. In the −2 charge state, the energy differences are larger. The third-best structure is C1(3) in the BS10–147 state. It is 16 kJ/mol less stable than the best one and for this structure, it is not favorable to form the C–C bond (such a structure is 28 kJ/mol higher in energy). The fourth-best structure is C1(5) in the BS10–147 state (still without any C–C bond), which is 20 kJ/mol less stable than the best one. The most stable structure with an O atom protonated is O1(3), 40 kJ/mol less stable than the C2(3)-C_*cis*_ structure, whereas in the −4 charge state, O1(3) and O2(3) are degenerate, 83 kJ/mol less stable than the best structure.

For seven of the structures, we studied the barrier for the C–C bond formation. For the five for which a stable structure with the C–C bond was found (including the four best structures), the barriers were rather small, 20–67 kJ/mol.

Thus, our results indicate that the first proton will bind to a C atom, not a O atom. The best structure is C2(3)-C_*cis*_, but there are other structures rather close in energy, both with and without a C–C bond.

### Proton Transfers within the E_3_ State

At the beginning of the study of the E_3_ state, we just added an electron and a proton to the E_2_ state. Electrons are provided to the FeMo cluster from the Fe protein via the P-cluster^1^ and they move freely within the FeMo cluster. Two pathways have been suggested for the transfer of protons to the cluster. One involves His-195, which forms a hydrogen bond to S2B in the resting state of the protein and therefore is close to the CO-binding site. However, it has been suggested that His-195 can only provide one proton because rotation of the imidazole side chain is restricted in the protein.^50,51^ The other pathway ends at a water molecule that is close to homocitrate, S3B, S4B, and S5A.^51−54^ Several authors have studied proton transfer along this pathway and inside the FeMo cluster for various states in putative reaction mechanisms.^22,50,52,54^ The conclusion has been that individual proton transfers within the cluster have rather low barrier. However, sometimes certain protonation states act as thermodynamic sinks, making the net barrier prohibitively high.

**Figure 3:**
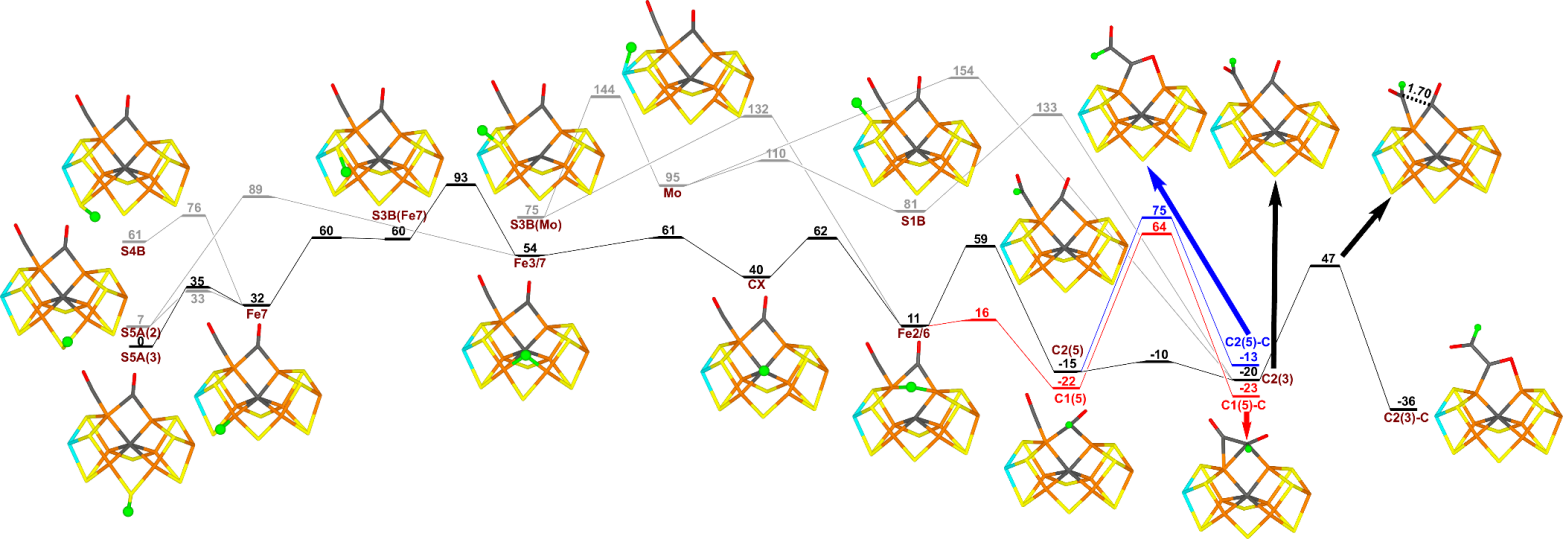
Relative energies (kJ/mol) for different protonation and transition states in the E_3_ state. To make the crowded right-hand part of the figure easier to interpret, two paths are shown in red and blue, respectively. The arrows point at the figures of the structures.

Therefore, we have performed a study of possible proton transfers within the FeMo cluster for the E_3_ state with two bound CO molecules, adding the proton either to S3B, S4B, or S5A, and then letting it move around the cluster (for a –4 charge of the QM system). The results are shown in Figure 3. It can be seen that among the cluster atoms, protonation of S5A is most favorable, being 60 and 61 kJ/mol lower in energy than protonation of S3B and S4B, and also 11–95 kJ/mol more favorable than protonation of other sites in the FeMo cluster (a proton bridging Fe2 and Fe6 being the second best). The individual activation barriers for proton transfer within the FeMo cluster are 7–82 kJ/mol, whereas proton transfers to CO show a variation from 5–59 kJ/mol. We included also the six best E_3_ substrate structures in this study and all of them are more stable than the best structure that has the proton in the FeMo cluster, viz., on S5A(3), by 13–36 kJ/mol.

As can be seen from Figure 3, the best proton-transfer path goes from S5A(3) via Fe7, S3B, Fe3/7, the central carbide (CX in Figure 3), and Fe2/6 to the terminal CO molecule, forming the C2(5) structure discussed in the previous section. The individual steps have small barriers, but counting from S5A(3), the barrier for the transfer of the proton from S3B(Fe7) to Fe3/7 is 93 kJ/mol, corresponding to a rate constant of 0.0005 s^–1^ (2 h^–1^; assuming a pre-exponential factor of 6.2 × 10^12^ s^–1^, i.e., slightly slower than the rate of CO reduction by Mo-nitrogenase, ∼7 h^–1^).^16^ It is most likely that the proton-transfer rate will be increased by proton tunnelling. Moreover, Siegbahn has argued that the proton-transfer within the cluster can be strongly sped up by the use of surrounding water molecules.^55,56^ The C2(5) structure can be converted to C2(3) by a minimal barrier (5 kJ/mol), in which the C–C bond can form by a barrier of 67 kJ/mol, giving the most stable E_3_ structure, C2(3)-C_*cis*_. The other E_3_ structures can also form, but two of the barriers are 87–91 kJ/mol (i.e., similar to that needed for the transfer of the proton from S5A(3) to the substrate). In conclusion, the proton-transfer barriers for CO-bound Mo-nitrogenase are similar to those observed for intermediates in the N_2_-reduction reaction,^22,50,52,54^ and it is unlikely that they limit the reaction rate of the protein. Therefore, we will not study the proton-transfer reactions for the other E_*n*_ states in this study.

### E_4_ Structures

**Figure 4:**
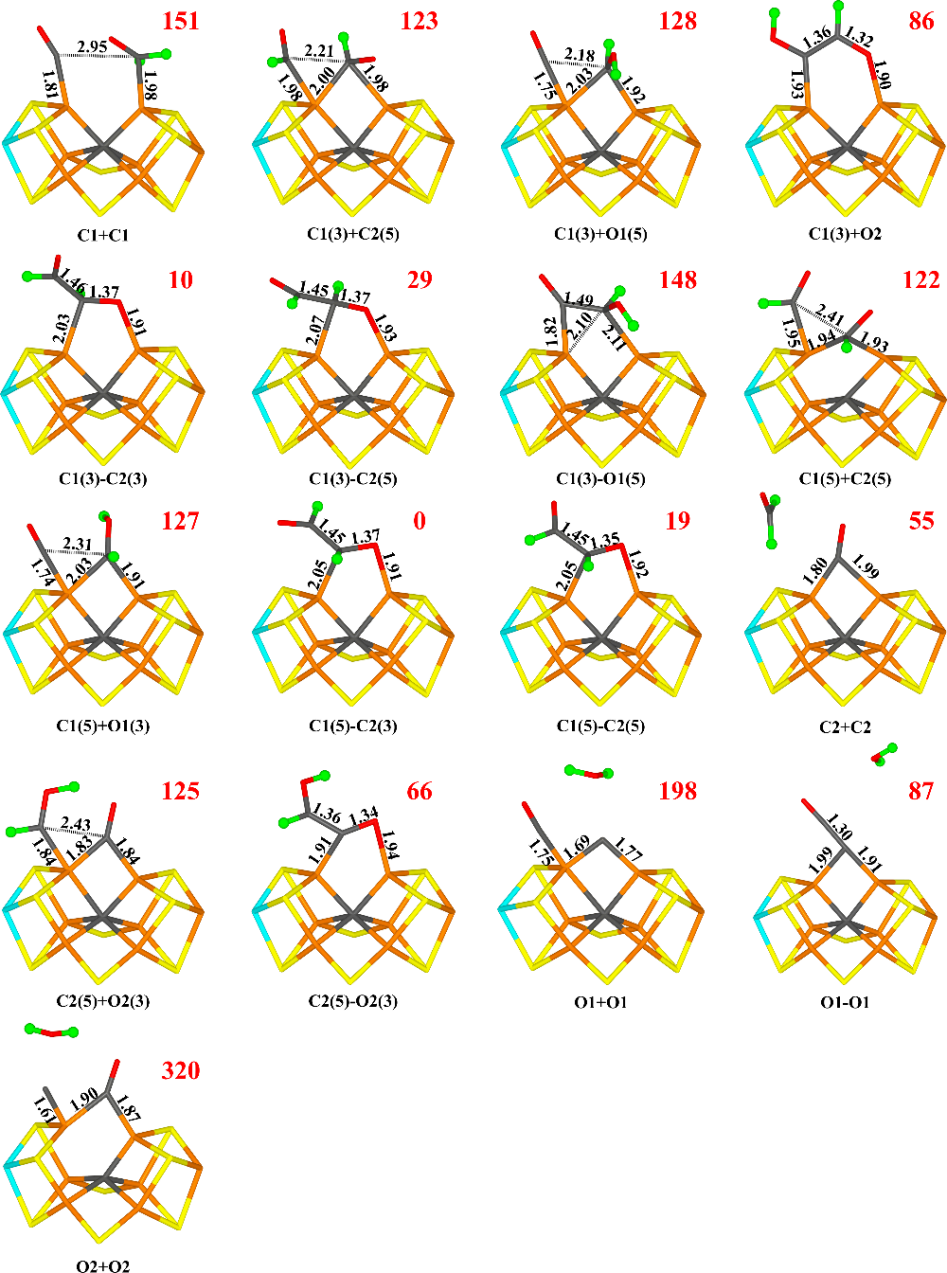
Structures considered for the E_4_ state, showing those obtained for the −4 charge state. Relative energies (kJ/mol) for the −4 charge states are indicated for each structure.

Next, we added an electron and a proton to the FeMo cluster to obtain structures at the E_4_ level. We studied 12 different protonation states, most of them both with and without a C1–C2 bond. In total, 17 different structures were found. They are denoted in a similar way as for the E_3_ structures (i.e., giving the name of the two protonated atoms with the direction of the proton, joined by either “–” or “+”, indicating a C1–C2 bond or not (e.g., C1(3)-C2(3), cf. Figure 4). We first optimized all structures in the BS10–147 state. We tested both the doublet and quartet states and found that the quartet was lowest, but the two spin states are often close in energy. Subsequently, after obtaining the lowest energy species, a comprehensive study of all BS states was carried for the best structure. BS7–247 was found to be lowest in energy. Therefore, calculations with the BS7–247 state were carried out for all structures. We studied both the −2 and −4 charge states. The relative energies are collected in Table 2.

**Table 2:** Relative Energies (kJ/mol) of the Various Structures Studied for the E_4_ State, Using Two Different Total Charges of the QM Region, −4 or −2^a^

Structure	–4	–2
C1+C1	151	117
C1(3)+C2(5)	123	129
C1(3)+O1(5)	128	62
C1(3)+O2	86	62
C1(3)-C2(3)	10	0
C1(3)-C2(5)	29	15
C1(3)-O1(5)	148	98
C1(5)+C2(5)	122	117
C1(5)+O1(3)	127	47
C1(5)-C2(3)	0	3^c^
C1(5)-C2(5)	19	31
C2+C2	55	37
C2(5)+O2(3)	125^b^	67
C2(5)-O2(3)	66	48
O1+O1	198^b^	324^b^
O1–O1	87	94
O2+O2	320^b^	376^b^

a All structures were studied in the quartet BS7-247 state, if not otherwise stated.

b BS10–135.

c BS7–235.

With a charge of −4, the most favorable structure is C1(5)-C2(3) with a C1–C2 bond (i.e., *trans*-glyoxal, Figure 4) in the quartet BS7–247 state. O1 coordinates to Fe2 (1.91 Å), whereas C1 coordinates to Fe6 (2.05 Å; cf. Figure 4), but it is also rather close to Fe2, 2.50 Å. C2 and O2 point away from the FeMo cluster and the C1–C2 bond length is 1.45 Å. The structure is stabilized by hydrogen bonds between O1 and HE2 of His-195 (1.67 Å) and between O2 and HE2 of Gln-191 (2.19 Å).

The corresponding *cis*-glyoxal structure, C1(3)-C2(3), still with BS7–247, is only 10 kJ/mol less stable and is actually 5 kJ/mol more stable in the −2 charge state. The structures are quite similar to Fe2–O1, Fe6–C1, Fe2–C1 and C1–C2 distances of 1.91, 2.03, 2.66, and 1.46 Å. It still has a hydrogen bond between O1 and HE2 of His-195 (1.72 Å), but O2 does not form any hydrogen bond. However, in the −2 charge state, O1 bridges Fe2 and Fe6 with Fe2–O1 and Fe6–O1 distances of 2.01 and 2.05 Å, whereas the Fe6–C1 distance is 2.00 Å. The other two conformations of glyoxal (C1(3)-C2(5) and C1(5)-C2(5)) are 15–31 kJ/mol less stable than the best structure. The former is in the *trans* conformation and latter is in the *cis* conformation.

Other structures are appreciably less stable, especially those with two protons on the same O atom. Thus, the formation of water is not favorable in this step. The formation of formaldehyde (HCHO, i.e., C1+C1 or C2+C2) is also unfavorable by at least 37–55 kJ/mol for the two charge states. For most structures, it is favorable to form the C1–C2 bond (C1(3)-O1(5) is the only exception when both structures are obtained). Thus, we conclude that the E_4_ structure involves glyoxal with a C1–C2 bond.

For nine structures, we studied the barrier for the formation of the C1–C2 bond. For the seven structures that gave stable C–C bonds, the barriers are quite low, 4–48 kJ/mol. For the best two structures, the barriers are only 4–5 kJ/mol. For the most stable structures in the −4 charge state, no corresponding structures with a broken C–C bond were found.

### E_5_ Structures

**Figure 5:**
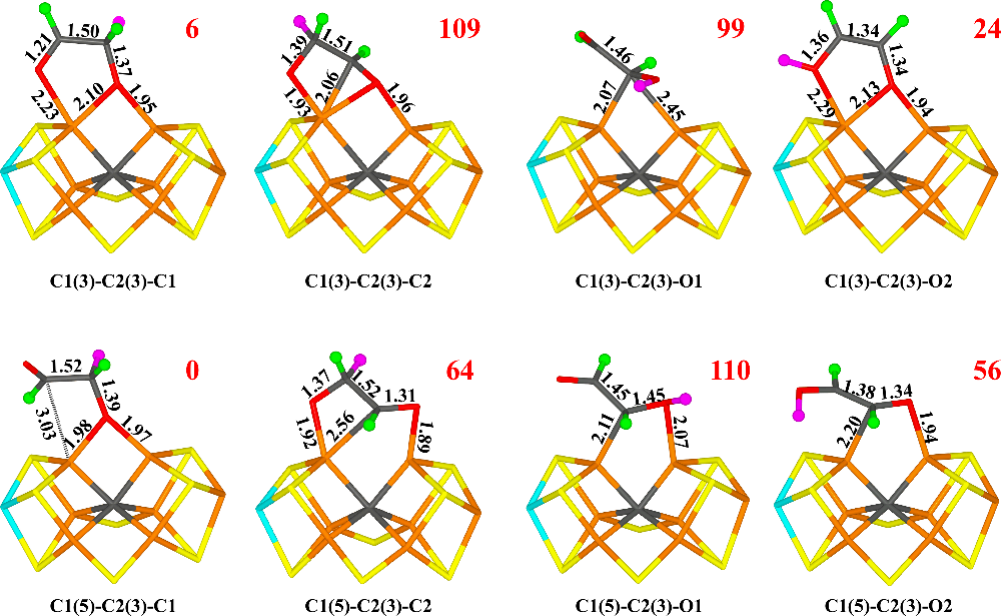
Structures considered for the E_5_ state, showing those obtained for the −4 charge state, except for C1(3)-C2(3)-C1 (which is the best structure for the −2 charge state). The latest added proton is shown in magenta, the other two as green balls. Relative energies (kJ/mol) for the −4 charge states are indicated for each structure.

Adding a proton and an electron to the previous structures gives intermediates at the E_5_ level. We started from the two best E_4_ structures, C1(3)-C2(3) and C1(5)-C2(3) (i.e., *cis*- or *trans*-glyoxal with a formed C1–C2 bond), because they were close in energy (within 3–10 kJ/mol for the two charge states). We tried to add a proton to all four C or O atoms of the substrate. As usual, we first studied the BS10–147 state, then did a comprehensive study of all BS states and three spin states, *S* = 0, 1 and 2, for the most stable structure, and finally reoptimized all structures with the lowest spin and BS state. The results are collected in Table 3 and Figure 5.

**Table 3:** Relative Energies (kJ/mol) of the Various Structures Studied for the E_5_ State, Using Two Different Total Charges of the QM Region, −4 or −2^a^

Structure	–4	–2
C1(3)-C2(3)-C1	6	0
C1(3)-C2(3)-C2	109	144
C1(3)-C2(3)-O1	99	128
C1(3)-C2(3)-O2	24	29
C1(5)-C2(3)-C1	0	22
C1(5)-C2(3)-C2	64^b^	135^b^
C1(5)-C2(3)-O1	110	120
C1(5)-C2(3)-O2	56	84

a All structures were studied in the quintet BS7-247 state, if not otherwise stated.

b BS-147.

Again, we find differences between the two charge states. For the −4 state, the most favorable structure is C1(5)-C2(3)-C1 (i.e., deprotonated *trans*-glycolaldehyde, OCHCH_2_O^–^) in the quintet BS7–247 state. The deprotonated alcohol O1 atom bridges Fe2 and Fe6 with O1–Fe2 and O1–Fe6 distances of 1.97 and 1.98 Å. The aldehyde group points toward homocitrate with a Fe6–C2 distance of 3.02 Å. It has a weak hydrogen bond between O1 and HE2 of His-195 (2.59 Å), but O2 does not form any hydrogen bond. There is essentially no spin on the substrate.

On the other hand, for the −2 state the most favorable structure is C1(3)-C2(3)-C1 (i.e., deprotonated *cis*-glycolaldehyde). The deprotonated alcohol O1 atom still bridges Fe2 and Fe6 with O1–Fe2 and O1–Fe6 distances of 1.95 and 2.04 Å, but the aldehyde O2 atom also forms a bond to Fe6 with a O2–Fe6 distance of 2.18 Å. It has still a weak hydrogen bond between O1 and HE2 of His-195 (2.44 Å). This structure is 6 kJ/mol less stable than C1(5)-C2(3)-C1 for the −4 charge state, whereas the opposite is true for the −2 state by 22 kJ/mol. Other structures are at least 24–29 kJ/mol less stable. Thus, the E_5_ structure most likely contains a deprotonated glycolaldehyde.

### E_6_ Structures

**Figure 6:**
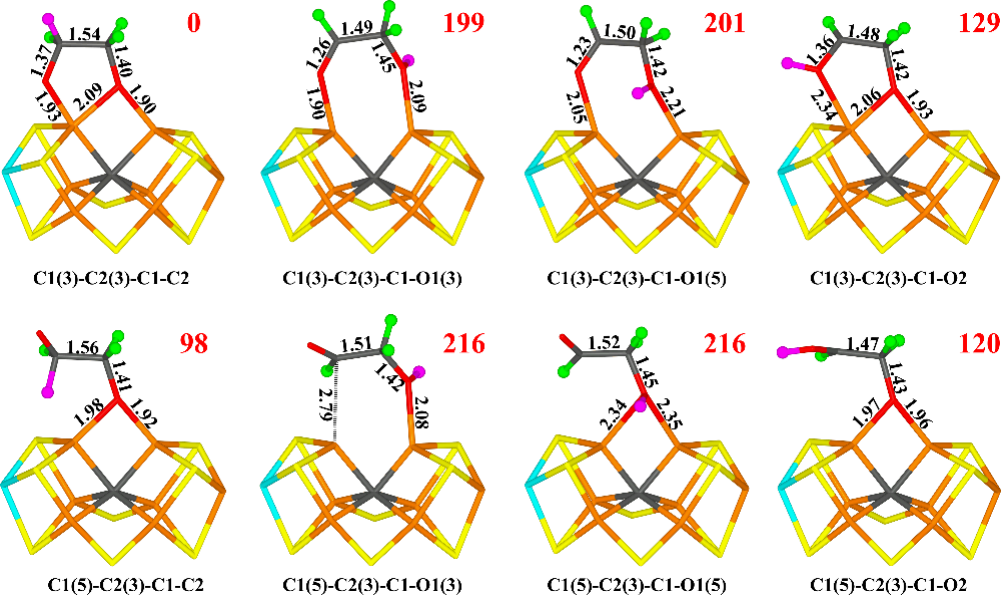
Structures considered for the E_6_ state, showing those obtained for the −4 charge state. Relative energies (kJ/mol) for the −4 charge states are indicated for each structure.

After adding yet another proton and electron, we reach the E_6_-level intermediates. We started from the best two E_5_ structures in Figure 5 (C1(3)–C2(3)–C1 and C1(5)-C2(3)-C1) and tried to add the extra proton to C2, O1 or O2 (there are already two protons on C1). As usual, we first studied all structures with BS10–147 state, then did a comprehensive study of all BS states and the two spin states (*S* = 1/2 and 3/2) for the most stable structure, and finally reoptimized all structures with the best BS and spin state. The results are listed in Table 4 and Figure 6.

**Table 4:** Relative Energies (kJ/mol) of the Various Structures Studied for the E_6_ State, Using Two Different Total Charges of the QM Region, −4 or −2^a^

Structure	–4	–2
C1(3)-C2(3)-C1-C2	0	0
C1(3)-C2(3)-C1-O1(3)	199	94
C1(3)-C2(3)-C1-O1(5)	201^b^	97
C1(3)-C2(3)-C1-O2	129	162
C1(5)-C2(3)-C1-C2	98	143
C1(5)-C2(3)-C1-O1(3)	216	146
C1(5)-C2(3)-C1-O1(5)	216	134
C1(5)-C2(3)-C1-O2	120	56

a All structures were studied in the quartet BS10-135 state, if not otherwise stated.

b BS-147.

The best structure for both charge states is C1(3)-C2(3)-C1-C2 (i.e., doubly deprotonated ethylene glycol, ^–^OCH_2_CH_2_O^–^), in the quartet BS10–135 state. As can be seen in Figure 6, O1 bridges Fe2 and Fe6, whereas O2 binds to Fe6, with Fe2–O1, Fe6–O1, and Fe6–O2 distances of 1.90, 2.09, and 1.93 Å or 1.88, 1.97, and 1.84 Å for the −4 and −2 charge states, respectively. The C1–O1 distance is 1.40–1.42 Å, the C2–O2 distance is 1.37–1.39 Å, and the C–C distance is 1.53–1.54 Å. It has a weak hydrogen bond between O1 and HE2 of His-195 (2.36–2.47 Å). The substrate has a significant radical character, with a net spin population of 0.28 and 0.37 for the −4 and −2 charge states, respectively, mainly on the two O atoms. Thus, the substrate has formally abstracted almost two electrons from the cluster, reaching the alcohol oxidation state level.

From the best structure E_5_ structure at the −4 charge state (C1(5)-C2(3)-C1), a similar structure can be obtained after protonation of C2, but O2 does not coordinate to Fe6 but instead points away from the cluster (C1(5)-C2(3)-C1-C2). Such a structure is 98 kJ/mol less stable than the structure obtained from C1(3)-C2(3) (in the −4 state). However, it can be converted to the other structure by a rotation around the C1–C2 bond, passing an activation energy of only 37 kJ/mol. Therefore, even if the two charge states differed at the E_5_ level, they converge again to the same structure at the E_6_ level.

Other structures are at least 94–129 kJ/mol higher in energy for the two charge states. We tried to cleave the C1–O1 bond or C2–O2 bond, but such reactions are strongly uphill. Thus, the C_2_H_4_ unit is formed in this step, but it cannot dissociate, because it is still too oxidized.

### E_7_ Structures

**Figure 7:**
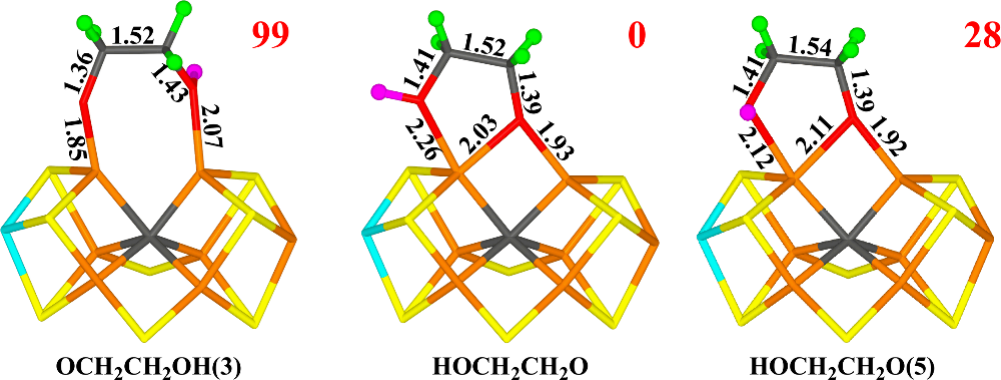
Structures considered for the E_7_ state, showing those obtained for the −4 charge state. Relative energies (kJ/mol) for the −4 charge states are indicated for each structure.

Next, we added an electron and a proton to either O1 or O2 of the best E_6_ structure in Figure 6 (C1 and C2 are already doubly protonated). The results are listed in Table 5 and shown Figure 7. The best structure has the H atom bound to O2 (HOCH_2_CH_2_O, i.e., singly protonated ethylene glycol) in the quintet BS7–247 state. As can be seen in Figure 7, the deprotonated O2 atom still bridges Fe2 and Fe6, with Fe–O distances of 1.92–1.93 and 2.00–2.03 Å, whereas the protonated O1 atom binds to Fe6 with a Fe–O distance of 2.11–2.26 Å (for the two charge states). The C–C bond is 1.52 Å and the two C–O bonds are 1.39–1.42 Å. O1 receives a weak hydrogen bond from His-195 (2.50–2.62 Å), whereas the hydrogen on O2 donates a hydrogen bond to homocitrate (1.63–1.73 Å). The other two structures are 28–118 kJ/mol less stable. We tried to cleave the C1–O1 bond and C2–O2 bond, but the activation energies are still prohibitively high.

**Table 5:** Relative Energies (kJ/mol) of the Various Structures Studied for the E_7_ State, Using Two Different Total Charges of the QM Region, −4 or −2^a^

Structure	–4	–2
OCH_2_CH_2_OH(3)	99	86
OCH_2_CH_2_OH(5)		118
HOCH_2_CH_2_O	0	0
HOCH_2_CH_2_O(5)	28	18

a All structures were studied in the quintet BS7-247 state.

### E_8_ Structures

**Figure 8:**
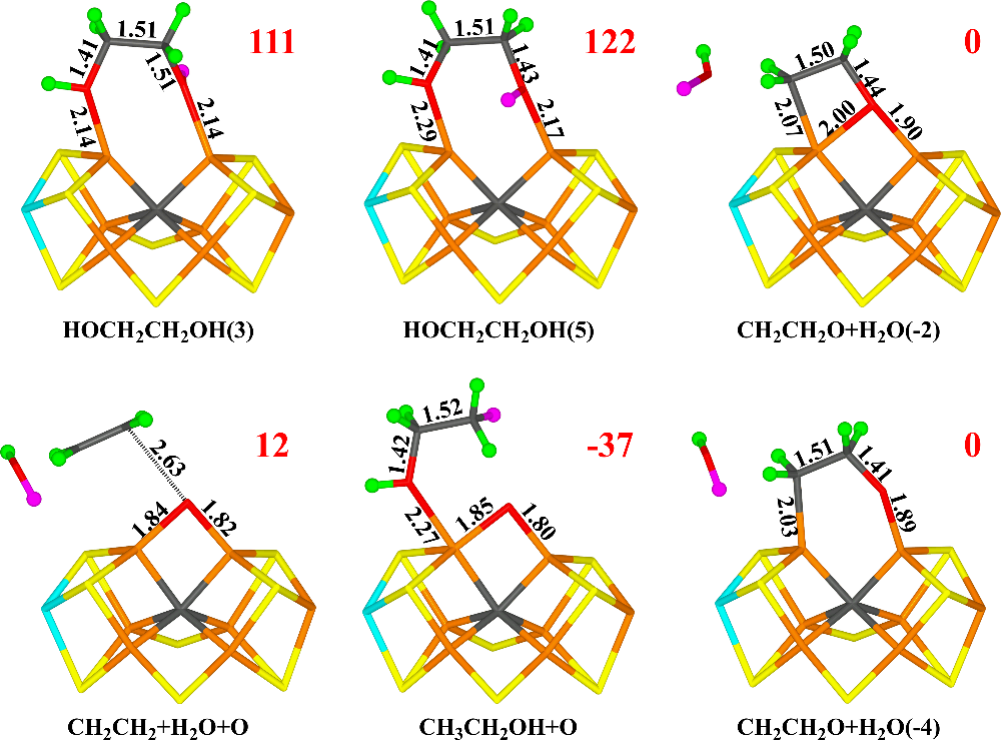
Structures considered for the E_8_ state, showing those obtained for the −4 charge state, except for CH_2_CH_2_O+H_2_O, which is shown in both charge states. Relative energies (kJ/mol) for the −4 charge states are indicated for each structure.

Next, we added an electron and a proton to O1 or O2 of the best E_7_ structure in Figure 7. The structures are listed in Table 6 and are shown Figure 8. The best structure has the proton on O2. This leads to cleavage of the C2–O2 bond, giving rise to a doubly deprotonated ethanol molecule (^−^CH_2_CH_2_O^–^) and a water molecule that dissociates from the FeMo cluster and forms a hydrogen bond to Glu-380 and to S1B (Figure 8). The two charge states give somewhat different structures. In the −4 state (for which BS7–346 is most stable), O1 binds to Fe2, with a Fe2–O1 bond length of 1.89 Å, and it also forms a very strong hydrogen bond to His-195, with a HE2–O1 distance of 1.52 Å. C2 binds to Fe6 with a Fe6–C2 bond length of 2.04 Å. The spin population on the ^–^CH_2_CH_2_O^–^ group is rather low, 0.14 *e*.

**Table 6:** Relative Energies (kJ/mol) of the Various Structures Studied for the E_8_ State, Using Two Different Total Charges of the QM Region, −4 or −2^a^

Structure	–4	–2
HOCH_2_CH_2_OH(3)	111^b^	33^c^
HOCH_2_CH_2_OH(5)	122^b^	4^d^
CH_2_CH_2_O+H_2_O	0	0^e^
CH_2_CH_2_+H_2_O+O	12	68
CH_3_CH_2_OH+O	–37	19^b^

a All structures were studied in the quartet BS7-346 state.

b Doublet BS10–147.

c Doublet BS10–146.

d Quartet BS10–125.

e Quartet BS7–235.

In the −2 charge state (for which BS7–235 is most stable), the alcohol oxygen atom O1 bridges Fe2 and Fe6 with Fe2–O1 and Fe6–O1 distances of 1.91 and 2.03 Å, whereas C2 coordinates to Fe6 with a Fe6–C2 distance of 2.05 Å. The O1–HE2 hydrogen bond is appreciably longer, 2.22 Å. The ^–^CH_2_CH_2_O^–^ group has an appreciably larger radical character in this structure, 0.35 *e*. We have confirmed that these two structures are caused by differences in the charge (and BS) state and are not only two local minima.

We tried to cleave also the C1–O1 bond for this structure. For the −2 charge state, the energy barrier is prohibitively high, 134 kJ/mol, and the product is 68 kJ/mol less stable than the best state. However, for the −4 charge state, the barrier is only 79 kJ/mol, which could be passed. The product involves an ethylene molecule that dissociates from the cluster (C–C bond length 1.34 Å) and O^2–^ bridging Fe2 and Fe6 (the Fe2–O1 and Fe6–O1 bonds are 1.82 and 1.84 Å; O1 accepts a hydrogen bond from His-195, 2.21 Å). The product is 12 kJ/mol less stable than the reactant state, but this is most likely compensated by the entropy gain of the dissociating ethylene molecule (typically estimated to ∼45 kJ/mol).^57,58^

In the −4 charge state, the other two structures (HOCH_2_CH_2_OH(3) and HOCH_2_CH_2_OH(5), i.e., neutral ethylene glycol with the proton pointing in different directions) are 111–122 kJ/mol less stable. However, in the −2 charge state, the HOCH_2_CH_2_OH(5) structure is only 4 kJ/mol less stable than the best structure (but this difference would most likely be increased by the gain of entropy from the released water molecule). The O1 atom bridges Fe2 and Fe6 (the Fe–O1 distances are 2.14 and 2.42 Å) and O2 binds to Fe6 (Fe–O2 = 2.16 Å). The C1–C2 bond distance is 1.52 Å, the C1–O1 distance is 1.46 Å, and the C2–O2 bond distance is 1.41 Å. O1 receives a hydrogen bond from HE2 of His-195 (2.17 Å), whereas the proton on O2 donates a hydrogen bond to homocitrate (1.61 Å). There is only minor spin on the ligand, 0.05 *e*. The HOCH_2_CH_2_OH(3) structure (with the proton on O1 pointing in the opposite direction) is 33 kJ/mol higher in energy. The C1–O1 bond can be cleaved in the latter structure, but the barrier is high, 95 kJ/mol and the product (CH_2_CH_2_OH and OH^–^ bridging Fe2 and Fe6) is 66 kJ/mol less stable than the starting structure. The C2–O2 bond cannot be directly cleaved (barrier >300 kJ/mol). Moving the proton on O1 to C1, a structure with CH_3_CH_2_OH bound to Fe6 and O^2–^ bridging Fe2 and Fe6 can be obtained, which is only 19 kJ/mol less stable than the O2 structure in the −2 charge state, but the activation energy is prohibitively high, 297 kJ/mol. However, in the −4 charge state, such a structure is 37 kJ/mol more stable than the O2 structure, although this will be offset by the entropy of the dissociating water molecule. It has Fe–O1 bonds of 1.79 and 1.85 Å and a Fe6–O2 bond length of 2.27 Å.

### E_9_ and E_10_ Structures for the Charge −4 State

As we saw in the previous section, the reaction paths of the charge −2 and −4 states split up at the E_8_ level. For the −4 charge state, the ethylene product can form in the E_8_ state and the E_9_ and E_10_ states are needed only to protonate the bridging O^2–^ ion to water (which may exchange with SH_2_ to regenerate the resting E_0_ state or with two CO molecules to regenerate the E_2_ CO-bound state). These structures are described in this section. We assume that both formed molecules (water and ethylene) have diffused away from the active site.

**Figure 9:**
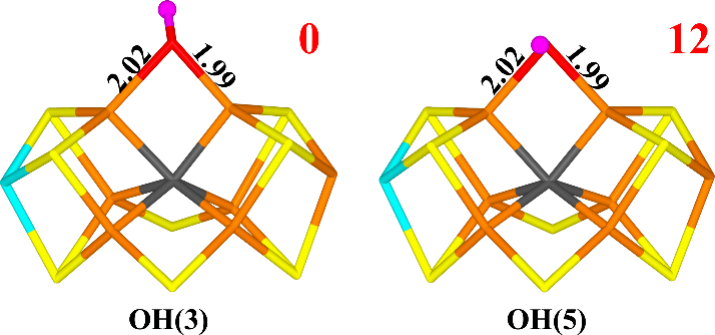
Structure considered for the E_9_ state for models with a net charge of −4. Relative energies (kJ/mol) are indicated for each structure.

Thus, we removed water and ethylene and added an electron and a proton to O1, pointing toward S3A. The OH^–^ group bridges Fe2 and Fe6 with Fe–O distances of 1.99 and 2.02 Å (Figure 9). O1 receives a hydrogen bond from His-195 (2.30 Å). There is almost no spin on the OH^–^ group (0.02 *e*). It is most stable in the sextet BS7–247 state. The structure with the proton on O1 pointing in the opposite direction (toward S5A) is 12 kJ/mol less stable (Table 7).

**Table 7:** Relative Energies (kJ/mol) of the Various Structures Studied for the E_9_ and E_10_ States with a Total Charge of the QM Region of −4^a^

Structure	Δ*E*
E_9_	
OH(3)	0
OH(5)	12
E_10_	
H_2_O(Fe2/6)	43
H_2_O(Fe6)	0

a All structures were studied in the quintet BS7-247 state for E_9_ and quartet BS7-235. Compared to the other structures in this study, it was assumed that H_2_O and C_2_H_4_ has dissociated from the protein.

**Figure 10:**
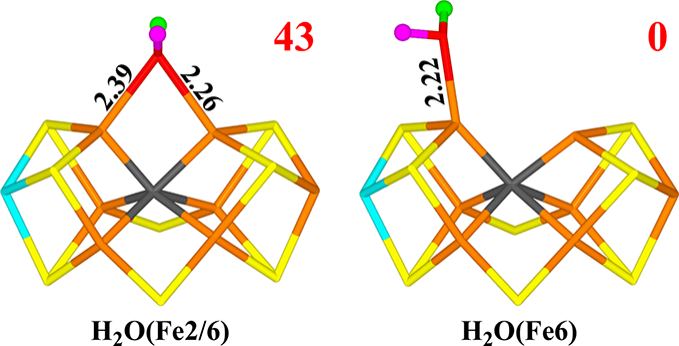
Structures considered for the E_10_ state for models with a net charge of −4. Relative energies (kJ/mol) are indicated for each structure.

Adding yet another electron and proton to the best structure gives the E_10_ state with a water molecule bridging Fe2 and Fe6 (Figure 10). It is most stable in the quartet BS7–235 state, in agreement with the E_0_ state of the enzyme.^21^ However, water can dissociate from Fe2 with essentially no barrier (1 kJ/mol). This gives a structure in which the water molecule binds only to Fe6 (Fe6–O bond length of 2.22 Å). It is still most stable in the quartet BS7–235 state and is 43 kJ/mol more stable than the bridging structure. One of the H atoms on water forms a hydrogen bond to homocitrate (1.95 Å).

### E_9_ and E_10_ Structures for the Charge −2 State

For the charge −2 state, the most stable E_8_ structure had ^–^CH_2_CH_2_O^–^ bound to Fe2 and Fe6 and water dissociated from the cluster (but ethylene could not form from this structure). However, a structure with HOCH_2_CH_2_OH bound to Fe2 and Fe6 was only 4 kJ/mol less stable. Therefore, we started from these two structures and added one electron and a proton to reach the E_9_ state.

**Figure 11:**
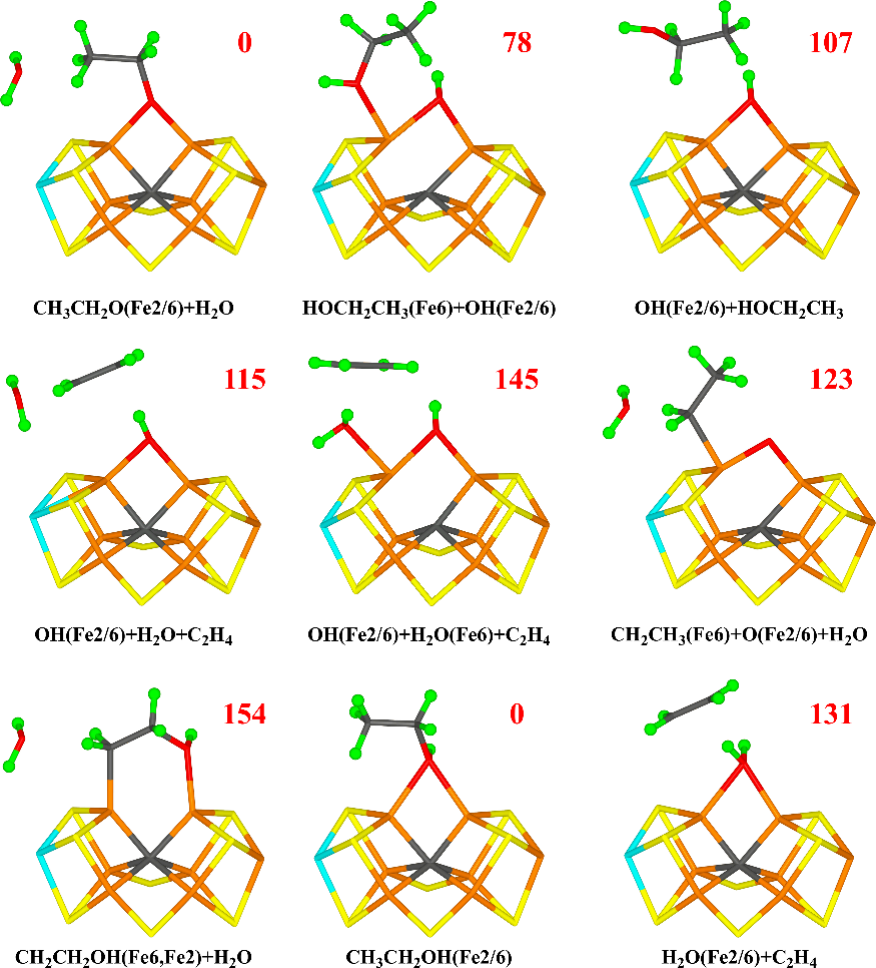
Structures considered for the E_9_ and E_10_ state for models with a net charge of −2. Relative energies (kJ/mol) are indicated for each structure.

We studied nine different structures, shown in Figure 11 and listed in Table 8. The most stable (CH_3_CH_2_O(Fe2/6)+H_2_O) has CH_3_CH_2_O^–^ (deprotonated ethanol) bound by the O atom to both Fe2 and Fe6 (Fe–O bond lengths of 1.91 and 1.95 Å) and water in the second coordination sphere, forming a hydrogen bond to Glu-380 (1.59 Å). O1 receives a weak hydrogen bond from His-195 (2.60 Å). It has essentially no spin on CH_3_CH_2_O^–^ (0.02 *e*). We tried to move a proton from the methyl group to the O1 atom (to form C_2_H_4_), but this had a prohibitively large barrier (∼300 kJ/mol).

**Table 8:** Relative Energies (kJ/mol) of the Various Structures Studied for the E_9_ and E_10_ States With a Total Charge of the QM Region of −2^a^

Structure	Δ*E*
E_9_	
CH_3_CH_2_O(Fe2/6)+H_2_O	0
HOCH_2_CH_3_(Fe6)+OH(Fe2/6)	78
OH(Fe2/6)+HOCH_2_CH_3_	107
OH(Fe2/6)+H_2_O+C_2_H_4_	115
OH(Fe2/6)+H_2_O(Fe6)+C_2_H_4_	145
OH(Fe2/6)+H_2_O(Fe6)+C_2_H_4_	161^b^
CH_2_CH_3_(Fe6)+O(Fe2/6)+H_2_O	123
CH_2_CH_2_OH(Fe6, Fe2)+H_2_O	154^c^
E_10_	
CH_3_CH_2_OH(Fe2/6)	0
H_2_O(Fe2/6)+C_2_H_4_	131

a All structures were studied in the quintet BS7-247 state for E_9_ and doublet BS10-147 for E_10_, if not otherwise stated.

b BS10–147.

c BS7–346.

The second-best structure (HOCH_2_CH_3_(Fe6)+OH(Fe2/6)) has OH^–^ bridging Fe2 and Fe6 (Fe–O bond lengths of 1.89 and 2.05 Å) and CH_3_CH_2_OH (ethanol) bound to Fe6 (Fe–O bond length of 2.13 Å). It is most stable in the quintet BS7–247 state, but it is 78 kJ/mol less stable than the best structure. Dissociating ethanol has a moderate barrier (56 kJ/mol) and is unfavorable by 29 kJ/mol, but this is probably reversed by entropy effects. Transferring a proton from the methyl group to the alcohol O atom has a very larger barrier (∼250 kJ/mol).

A structure with OH^–^ bridging Fe2 and Fe6 (with Fe–O bonds of 1.89 and 1.93 Å) and with both ethylene and water dissociated from the cluster is 115 kJ/mol less stable than the best structure (which can be partly offset by entropy). A structure with the water molecule bound to Fe6 (and OH^–^ still bridging) is 30 kJ/mol less stable. Both structures are most stable in the quintet BS7–247 state.

A structure with O^2–^ bridging Fe2 and Fe6, CH_2_CH_3_ binding to Fe6 and water dissociated from the cluster is 123 kJ/mol less stable than the best structure and another structure with ^–^CH_2_CH_2_OH binding to Fe2 (with O) and Fe6 (with C2) and a dissociated water is 154 kJ/mol less stable than the best state. In the latter structure, the C–O bond can easily be cleaved, to form C_2_H_4_ (activation barrier 60 kJ/mol), but such a reaction is unlikely because the reactant is so unstable.

Finally, we added an electron and a proton to the best E_9_ state (removing the water molecule), forming CH_3_CH_2_OH, (ethanol) with the O atom bridging Fe2 and Fe6 with Fe–O distances of 2.17 and 2.19 Å. We tried to move a proton from the methyl group to the O atom (initial distance 2.77 Å), but this had a high barrier (210 kJ/mol) and the product (ethylene dissociated from the cluster and water bridging Fe2 and Fe6) is 131 kJ/mol less stable than the starting structure. Consequently, we need to conclude that ethylene cannot be formed in the −2 charge state.

## Conclusions

We have performed a QM/MM study of the formation of ethylene by nitrogenase. We start from the crystal structure of CO-inhibited nitrogenase featuring two CO molecules binding to the FeMo cluster.^12^ We have successively added electrons and protons, systematically investigation all possible protonation states of the substrate molecules for each E_*n*_ state. We also investigate possible BS and spin states.

**Figure 12:**
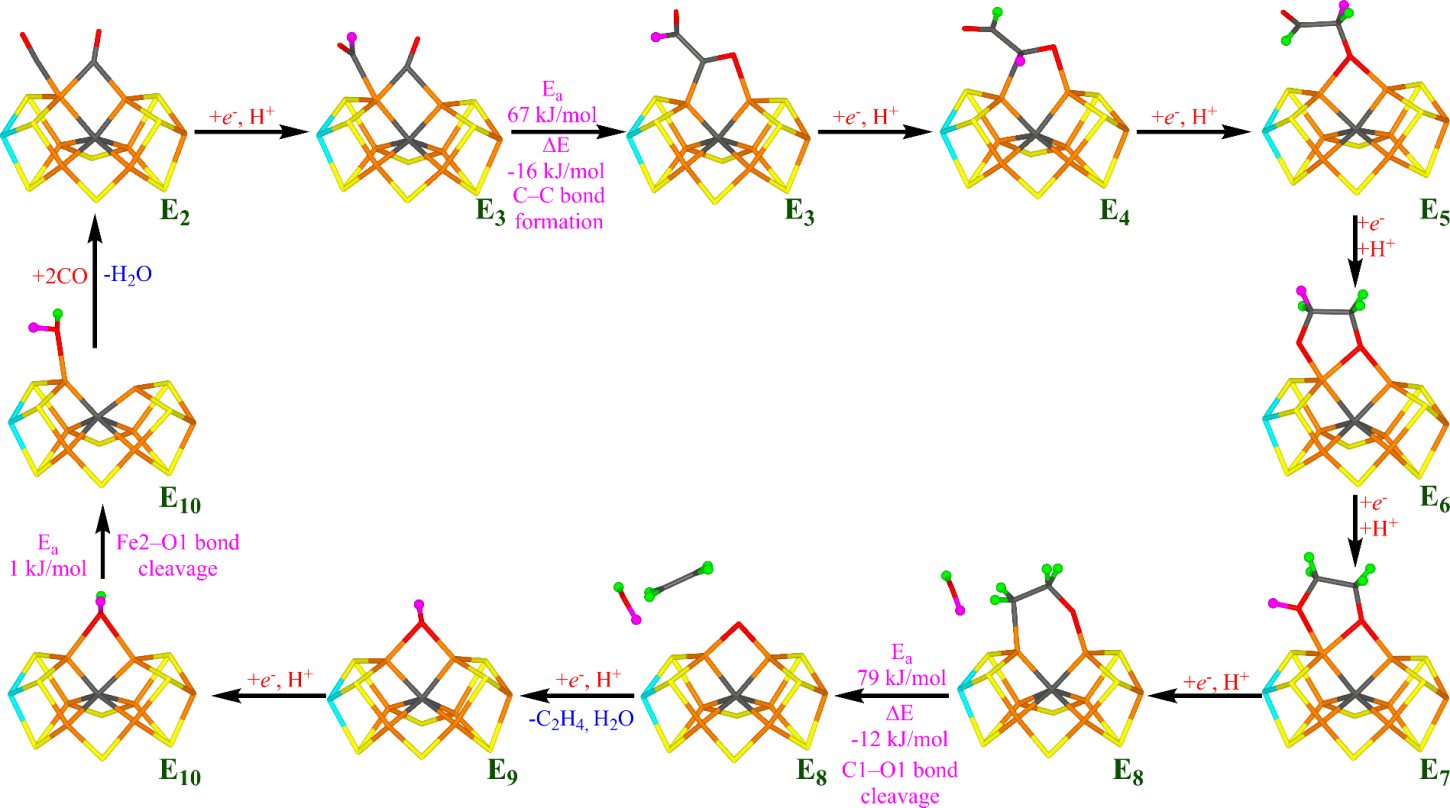
Suggested reaction mechanism for the CO reduction by nitrogenase. The proton added in each step is marked in magenta, whereas protons added in previous step are shown in green. The cluster needs to be in the −4 charge state (same charge as the E_0_ state).

Based on our calculations, we suggest the reaction mechanism shown in Figure 12. In this mechanism, the first four H atoms bind alternately to C1 and C2. Then, the following two H atoms bind to O2, leading to dissociation of water, and the last two protons bind to O1. Ethylene can form in the E_8_ state, but only if the cluster remains in the same charge state as E_0_ after binding of two CO molecules (implying a formal reduction of the cluster). The formation is partly driven by the entropy gain of dissociating ethylene. For some E_*n*_ levels, several protonation states and conformations are close in energy. It seems reasonable to assume that longer hydrocarbons may form by the binding of additional CO molecules before C_2_H_4_ is formed, but such reactions are not studied here.

For each new E_*n*_ state, we have assumed that a proton is added to the substrate. However, it is conceivable that the proton goes instead to the FeMo cluster. To check that possibility, we tested to instead add the proton to the S5A ion (directed toward S3A), because this is the most stable protonation state found in previous studies when S2B has dissociated, and it was also most favorable for the CO-bound structures for the E_3_ state in this study (cf. Figure 3). The results in Table S2 shows that it is always more favorable to add the proton to the substrate than to S5A by 36–171 kJ/mol. The only exception is the E_10_ state, for which the opposite is true by 29 kJ/mol. This does not affect the suggested mechanism in Figure 12, because we do not study the regeneration of the starting E_2_ structure in detail.

In previous studies,^22,50,52,54^ it has been shown that protons can reach the substrate from the proton channel, ending at a water molecule close to S3B, S4B, and S5A. In Figure 3, we showed that this is the case also for the E_3_ state in the present mechanism. Therefore, we did not do such a study also for the other states. However, for the E_4_ → E_5_ and E_5_ → E_6_ transitions in our suggested mechanism, there are quite large changes in the structure, indicating that it may be hard to transfer a proton from the FeMo cluster to the substrate in the proper position. Therefore, we performed detailed investigations of possible proton-transfer paths, starting from the optimum structure of the previous E_*n*_ state, adding an electron and a proton bridging Fe2 and Fe6. As can be seen in Figure S2, for both transitions, we could find low-energy paths for the transfer of the proton to the substrate in the optimum position (maximum barriers of 6 and 36 kJ/mol).

For 30 structures, we have performed a full investigation of all 35 BS states. This showed that there is a quite large variation in which BS state is most stable. In fact, 10 BS states are most stable for at least one structure: BS7–247 (11 structures); BS7–346 and BS10–135 (5 structures each); BS10–147 (3 structures); BS10–125 (2 structures); BS2–234, BS6–167, BS7–235, BS8–347, and BS10–146 (one structure each). Eight additional BS states are within 10 kJ/mol of the best BS state, BS3–124, BS6–156, BS6–157, BS8–345, BS9–137, BS9–145, BS10–127, and BS10–136, and three additional states are within 20 kJ/mol, BS3–134, BS8–236, and BS8–245. Thus, it is hard to select a small subset of BS states that always includes the best BS state.

Compared to the suggestions by Einsle and co-workers,^14,15^ our mechanism involves protons added to the substrate in each step, not a hydride ion added in every second step. Moreover, the C–C bond forms early in the mechanism, probably already in the E_3_ state, explaining why methane is not an important product. Finally, ethylene forms in the E_8_ state, without any need of β-elimination from an ethyl radical. On the other hand, several steps of our mechanism agree with the calculations of Dance,^19^ although he did not suggest a full reaction mechanism.

The study offers a thorough analysis of the nitrogenase mechanism, covering the primary binding of CO molecules to the ultimate E_10_ structures. It provides understanding on the reaction stages, C_2_H_4_ formation, the significance of broken symmetry and spin states, and the binding of protons to carbon atoms. It also establishes a robust foundation for future research and highlights the importance of ongoing investigations to fully understand the complexity and implications of the nitrogenase mechanism.

## Supporting Information Available

Key bond distances and optimized structures obtained by different functionals for the (CO)_2_-bound E_2_ state compared with the crystal structure, relative energies of the best structure for each E_*n*_ state and a state with the added proton on S5A, possible paths for the transfer of a proton bridging Fe2/6 to the optimum position on the substrate for the E_5_ and E_6_ states (PDF)
Coordinates for optimized structures and transition states (ZIP)
