# Supplementary material for: Reaction Mechanism for CO Reduction by Mo-Nitrogenase Studied by QM/MM
Source: Inorg Chem. 2024 Aug 14;63(34):15951–63. doi: 10.1021/acs.inorgchem.4c02323 (PMC11351180; doi:10.1021/acs.inorgchem.4c02323)

Supporting Information

Reaction mechanism for CO reduction by Mo-nitrogenase studied by QM/MM

Hao Jiang and Ulf Ryde *

Department of Computational Chemistry, Lund University, Chemical Centre,

P. O. Box 124, SE-221 00 Lund, Sweden

Correspondence to Ulf Ryde, E-mail: Ulf.Ryde@compchem.lu.se,

Tel: +46 – 46 2224502

2024-07-29

Table S1. Key bond distances (Å) calculated by different functionals for the $(CO)_2$-bound E_2 state compared with the crystal structure (PDB: 7JRF [12]). Two different charge states were considered viz. with a total charge (ch) for the QM system of –4 and –2. The structures were studied in the doublet BS10-147 state if not otherwise stated.

	Crystal	ch = –4	ch = –2				
	7JRF	r^2SCAN	TPSS	TPSSh	B3LYP	r^2SCAN	r^2SCAN[a]
Fe2–C1	1.93	1.86	1.83	1.85	1.92	1.90	1.87
Fe6–C1	1.92	1.89	1.97	1.95	1.88	1.87	1.89
Fe6–C2	2.03	1.74	1.77	1.79	1.80	1.76	1.75
Fe2–Fe6	2.53	2.49	2.49	2.51	2.49	2.50	2.50
Fe2–C	1.97	1.93	1.84	1.85	2.00	1.92	1.88
Fe6–C	2.06	2.15	2.14	2.03	2.10	2.12	2.14
C1–O1	0.95	1.21	1.19	1.18	1.18	1.19	1.19
C2–O2	1.12	1.16	1.16	1.15	1.14	1.15	1.15
O1–His195	2.84	2.59	2.64	2.68	2.66	2.65	2.64
O2–HCA	2.52	2.69	2.77	2.77	2.67	2.70	2.73
O2–Gln191	3.17	3.22	3.28	3.15	3.40	3.36	3.35

[a] Quartet BS10-135 state.

Table S2. Relative energy (kJ/mol) of the best structure for each E_n state in Tables 1–8 and a state with the added proton on S5A (directed towards S3A). A negative energy indicates that the latter state is more favourable. The inset figure shows the structure of the E_{10} state with S5A protonated (and OH^- bridging Fe2 and Fe6).

State	ΔE
E_3	36
E_4	138
E_5	171
E_6	163
E_7	123
E_8	72
E_9	76
E_{10}	-29

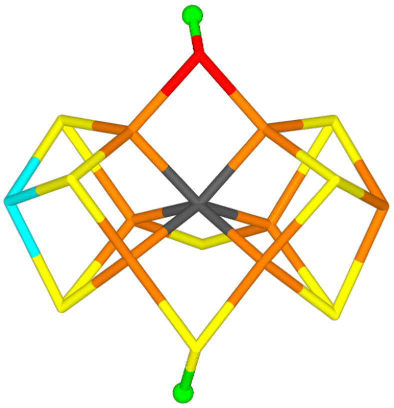

Figure S1. Optimised structures (charge –2, doublet BS10-147) compared with the crystal structure. (a) Crystal structure (PDB: 7JRF [12]). (b) Optimised structure with charge –4. (c) Optimised structure with charge –2. (d) Optimised structure with Gln-191 in a flipped conformation, observed in some crystal structures.[49]

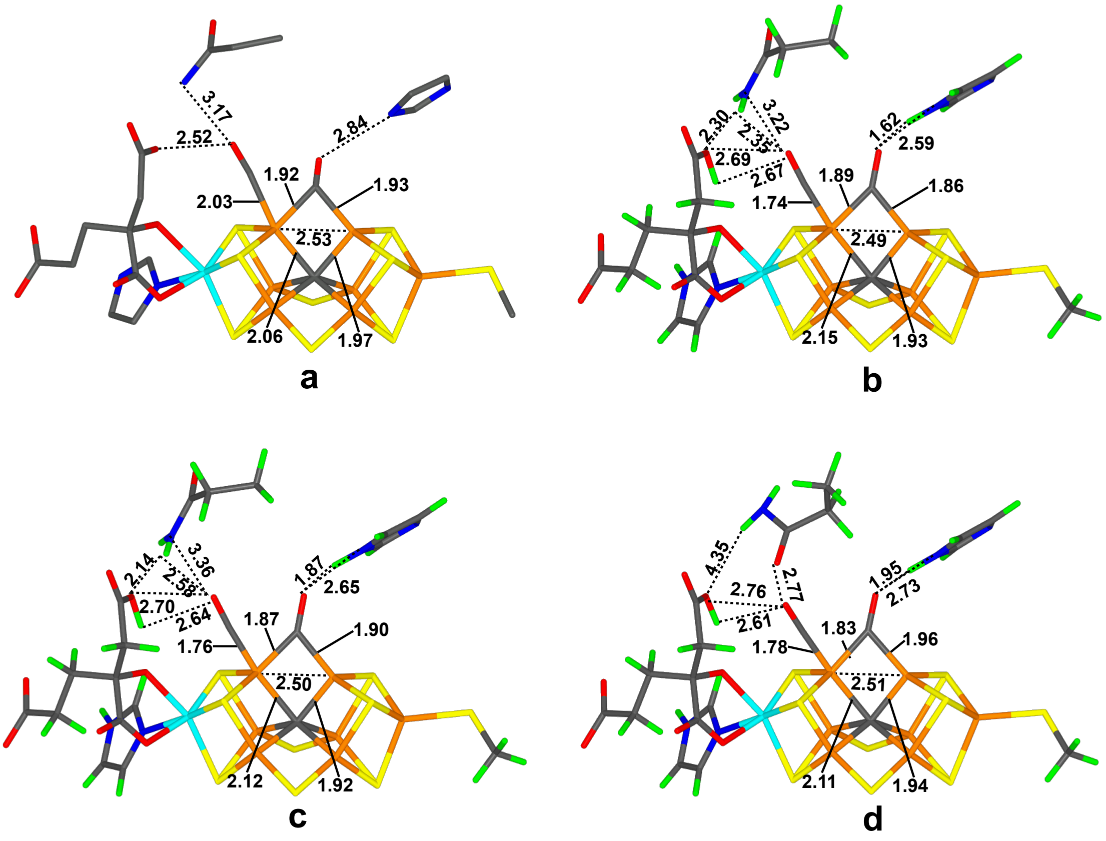

Figure S2. Possible paths for the transfer of a proton (hydride ion) bridging Fe2/6 (the proton is in both cases appreciably closer to Fe6 than to Fe2) to the optimum position on the substrate for the E_5 (top) and E_6 states (bottom). The calculations were started from the best E_4 and E_5 structures, respectively, adding an electron and a proton bridging Fe2/6. The results in Figure 3 shows that protons can move rather freely within the FeMo cluster, also to the Fe2/6 position. The C1→C1(5)-C2(3)-C1 transition in the E_5 state is essentially barrierless (less than 1 kJ/mol).

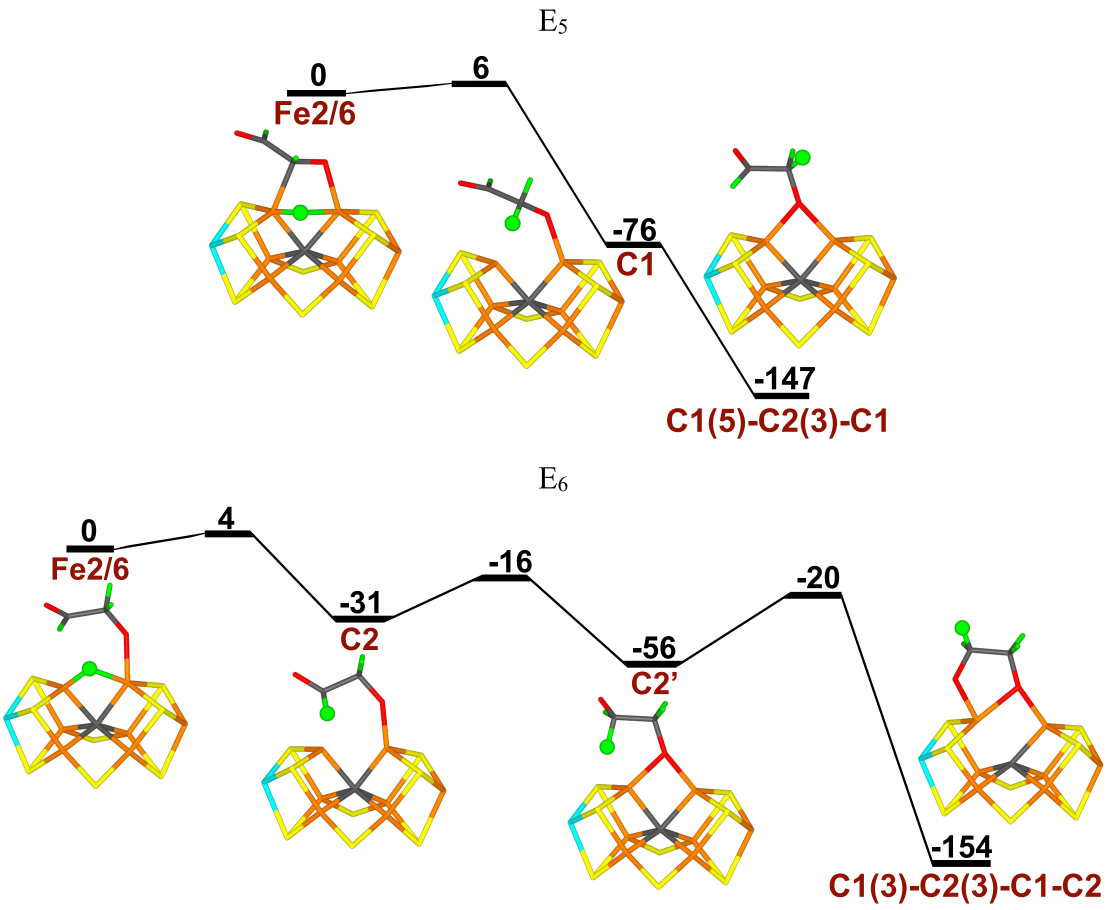

Supplement: Supplementary file 1 — ic4c02323_si_001.pdf [file ic4c02323_si_001.pdf]
